# A nasobiliary catheter landmark technique for endoscopic ultrasound-guided gallbladder drainage in patients with Billroth II anatomy: A case series

**DOI:** 10.1055/a-2913-7190

**Published:** 2026-07-29

**Authors:** Inho Lee, Sung Woo Ko, Seung Bae Yoon, Soyoon Oh, Jeongmi Lee, Hyehong Min, Ju Young Park

**Affiliations:** 1 Department of Internal Medicine 65644 Eunpyeong St. Mary’s Hospital, The Catholic University of KoreaSeoulKorea (the Republic of); 2 Outpatient Nursing Team 65644 Eunpyeong St. Mary’s Hospital, The Catholic University of KoreaSeoulKorea (the Republic of)

Endoscopic ultrasound-guided gallbladder drainage (EUS-GBD) is an established minimally invasive treatment for acute cholecystitis in patients unfit for surgery. 1​ However, duodenal access with a conventional oblique-viewing echoendoscope can be challenging in patients with Billroth II anatomy. Several reports have described the use of a forward-viewing echoendoscope to overcome this limitation. 2​ ​3​ Here, we present a nasobiliary catheter landmark technique that enables EUS-GBD using a conventional oblique-viewing echoendoscope in patients with Billroth II anatomy.

**Fig. 1:**
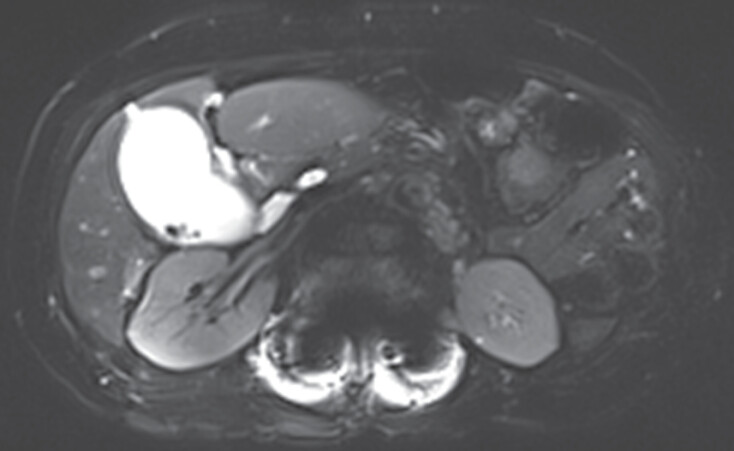
Magnetic resonance cholangiopancreatography demonstrating marked gallbladder distension and wall thickening consistent with acute cholecystitis.

An 82-year-old woman with a history of subtotal gastrectomy with Billroth II reconstruction for gastric cancer presented with acute cholecystitis. Magnetic resonance cholangiopancreatography demonstrated marked gallbladder distension and wall thickening ( Fig. 1 ). Because repeat surgery was considered high risk, EUS-GBD was planned.

**Fig. 2:**
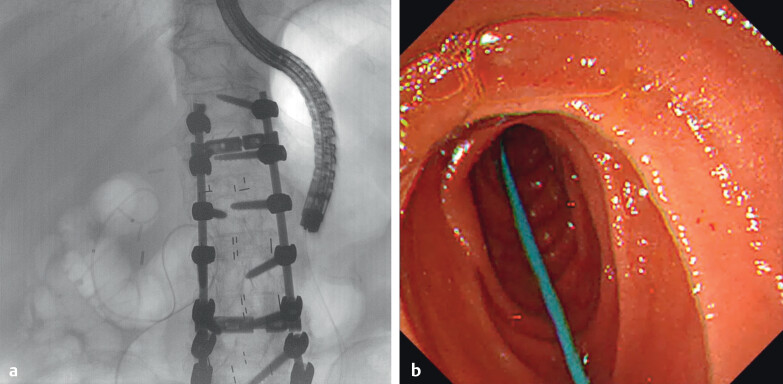
A nasobiliary catheter landmark technique for duodenal access. ( **a** ) A 5-Fr nasobiliary catheter is advanced deeply into the duodenum and left in situ after the withdrawal of the pediatric colonoscope. ( **b** ) A linear echoendoscope is advanced toward the duodenum using the nasobiliary catheter as an endoscopic landmark.

**Fig. 3:**
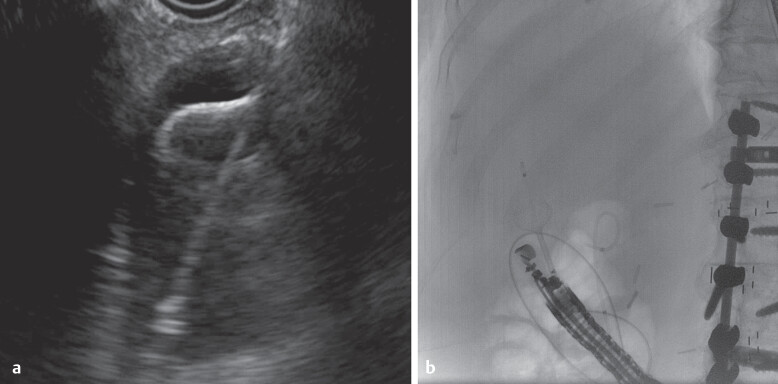
EUS-guided gallbladder drainage using a cautery-enhanced lumen-apposing metal stent. ( **a** ) An endoscopic ultrasound image showing a markedly distended gallbladder. ( **b** ) A fluoroscopic image demonstrating the successful deployment of the lumen-apposing metal stent.

**Fig. 4:**
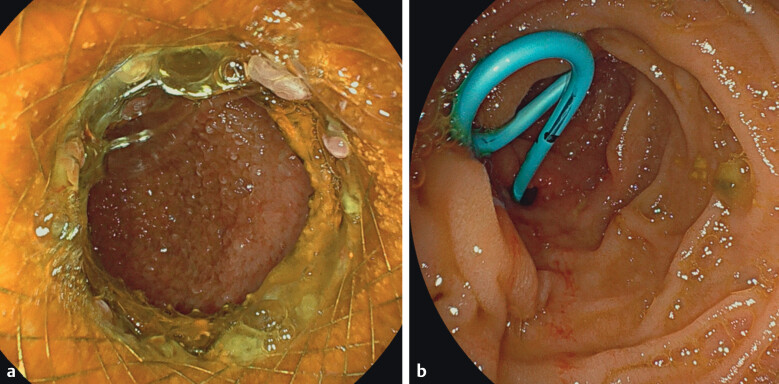
Peroral cholecystoscopy performed 4 weeks after EUS-guided gallbladder drainage. ( **a** ) Complete gallstone clearance is confirmed. ( **b** ) The lumen-apposing metal stent is exchanged for a double-pigtail plastic stent.

**Fig. 5:**
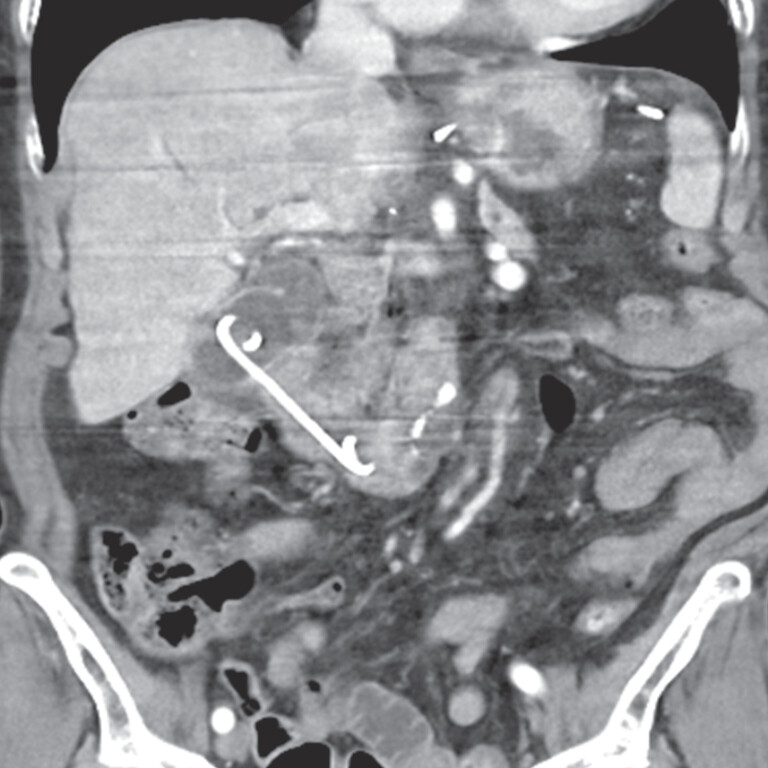
Eight-month follow-up computed tomography demonstrating the double-pigtail plastic stent in situ without recurrent cholecystitis.

A pediatric colonoscope (PCF-H290; Olympus, Tokyo, Japan) was advanced through the gastrojejunostomy into the afferent limb and duodenum. A 5-Fr nasobiliary catheter (A-Drain; A&A M.D., Seongnam-si, Korea) was advanced deeply into the duodenum, and the colonoscope was withdrawn. A linear echoendoscope (GF-UCT260; Olympus, Tokyo, Japan) was advanced toward the duodenum using the nasobiliary catheter as an endoscopic landmark ( Fig. 2 ). After visualization of a markedly distended gallbladder on EUS, EUS-GBD was performed using a 15×10 mm cautery-enhanced lumen-apposing metal stent (HOT AXIOS; Boston Scientific, Marlborough, Massachusetts, USA; Fig. 3 ; Video 1​ ). Four weeks later, peroral cholecystoscopy demonstrated complete gallstone clearance, and the lumen-apposing metal stent was exchanged for a double-pigtail plastic stent ( Fig. 4 ). Eight-month follow-up computed tomography demonstrated the plastic stent in situ without recurrent cholecystitis ( Fig. 5 ).

**Video 1** A nasobiliary catheter landmark technique for endoscopic ultrasound-guided gallbladder drainage using a conventional oblique-viewing echoendoscope in a patient with Billroth II anatomy.


Using this technique, EUS-GBD was successfully performed in five consecutive patients with Billroth II anatomy ( Table 1 ). The nasobiliary catheter landmark technique may facilitate EUS-guided GBD using a conventional oblique-viewing echoendoscope in patients with Billroth II anatomy.

**Table:** **Table 1** Clinical characteristics and procedural outcomes of five patients undergoing EUS-guided gallbladder drainage using the nasobiliary catheter landmark technique

Patient	Age/sex	PCF insertion-withdrawal time (min)	Echoendoscope insertion-LAMS deployment time (min)	Total procedure time (min)	Adverse events	Follow-up duration (mo)
1	82/F	9	13	22	No	15
2	82/M	8	4	12	No	9
3	87/M	14	9	23	No	10
4	86/M	9	11	20	No	7
5	95/M	9	12	21	No	1

Abbreviations: EUS, endoscopic ultrasound; LAMS, lumen-apposing metal stent; PCF, pediatric colonoscope.

Note: Technical success and clinical success were achieved in all five patients.

Endoscopy_UCTN_Code_TTT_1AS_2AK
